# Analogue of electromagnetically induced absorption with double absorption windows in a plasmonic system

**DOI:** 10.1371/journal.pone.0179609

**Published:** 2017-06-29

**Authors:** Nianfa Zhong, Qiaofeng Dai, Ruisheng Liang, Xianping Li, Xiaopei Tan, Xiaomeng Zhang, Zhongchao Wei, Faqiang Wang, Hongzhan Liu, Hongyun Meng

**Affiliations:** Guangzhou Key Laboratory for Special Fiber Photonic Devices, School of Information and Optoelectronic Science and Engineering, South China Normal University, Guangdong, China; Universitat Zurich, SWITZERLAND

## Abstract

We report the observation of an analog of double electromagnetically induced absorption (EIA) in a plasmonic system consisting of two disk resonators side-coupled to a discrete metal-insulator-metal (MIM) waveguide. The finite-difference time-domain (FDTD) simulation calculations show that two absorption windows are obtained and can be easily tuned by adjusting the parameters of the two resonance cavities. The consistence between the coupled-model theory and FDTD simulation results verify the feasibility of the proposed system. Since the scheme is easy to be fabricated, our proposed configuration may thus be applied to narrow-band filtering, absorptive switching, and absorber applications.

## Introduction

During last decades, mimicking the quantum phenomena in classical configurations has attracted enormous interest[1,2]. These analogs of quantum phenomena avoid tough experimental conditions and thus making it easier to realize practical applications. Among the quantum phenomena, electromagnetically induced transparency (EIT) is a quantum destructive interference phenomenon between the different excitation pathways to atomic levels and reduces light absorption over a narrow spectral region in a coherently driven atomic system[3]. A large body of research on analogs of EIT have been reported both in MIM waveguide and metamaterials[4–11]. In our group’s previous researches, we had studied analogues of electromagnetically induced transparency based on low-loss metamaterial[12] and metal-insulator–metal waveguide[13]. There are different approaches to generate EIT-like phenomenon in MIM waveguide based on surface plasmon polaritons (SPPs)[13–15], for example, by the near-field coupling and the phase coupling. Besides, by localized surface plasmon (LSP) modes based on metamaterials^4-6^, EIT-like phenomenon can be seen in cut wires, bilayer fish-scale structures, Fano resonators and so on. From above research reports, we could see a lot superb potential applications of the EIT-like effect, for instance, slow light, modulations, photonic switching.

As an opposite effect of EIT, electromagnetically induced absorption (EIA) is caused by a constructive interference of different excitation pathways and with an enhancement of absorbance[16]. In contrast with analogs of EIT, EIA-like phenomenon has only been sparsely investigated. For example, Zhang et al.[1] reported a classical analog of EIA in a three-resonator metasurface system and Tauberte et al.[17] observed the EIA-like phenomenon by employing the benefits of near-field coupling and retardation effects due to far field coupling. These kind of classical analogs of EIA showed its potential applications such as ultra-narrow band perfect absorbers [18], optical modulation, and absorptive switching[19].

In this study, we propose a plasmonic system consisting of a discrete stub-shaped MIM waveguide with dual side-coupled disk cavities to mimic the functionality of an atomic EIA system. By calculating the proposed structure with finite difference time-domain (FDTD) method, we obtained double EIA-like spectra. Besides, we introduced the coupled mode theory (CMT) based on transmission line theory to explain the EIA-like phenomenon theoretically. The absorption window can be adjusted by changing the geometrical parameters of cavities. This novel system is attractive since its form is simple and easy to fabricate. Our work provides a promising scheme for the realization of EIA-like spectra based on MIM waveguide structure.

## Simulation method and analysis of single-resonator-coupled system

**Fig 1:**
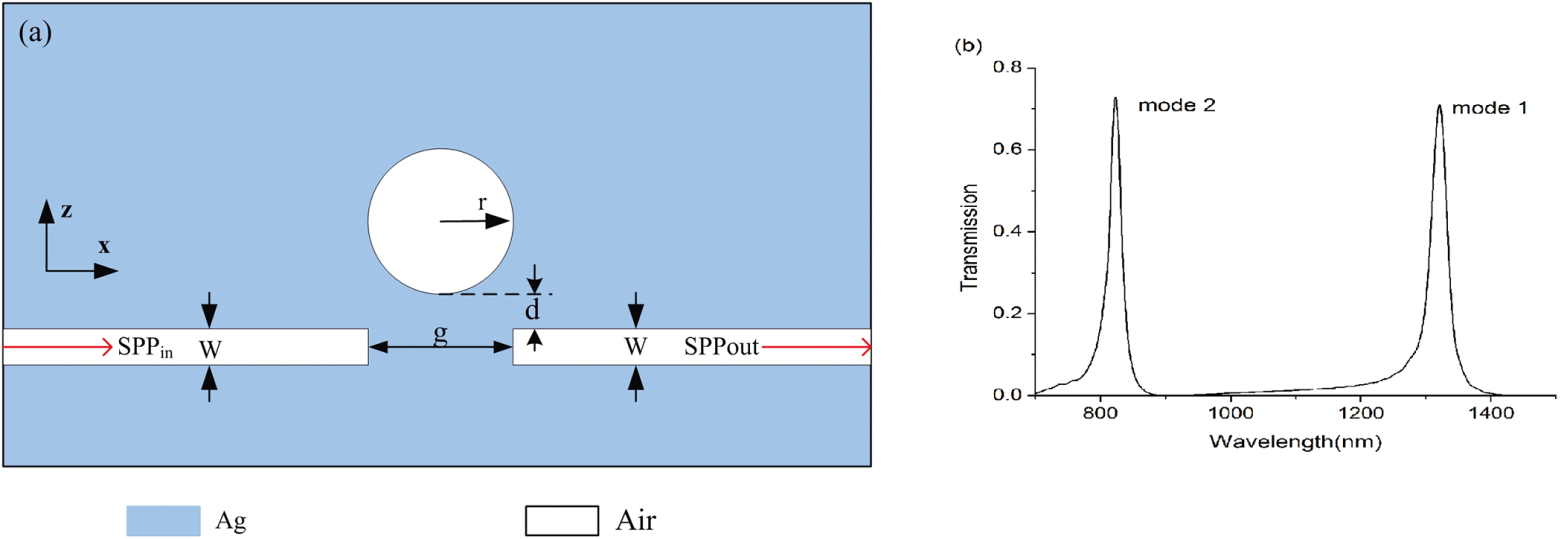
(a) Schematic of the single cavity structure. (b).Transmission spectra of the single cavity structure with W = 50nm, r = 345nm, g = 30nm, d = 12nm.

The schematic diagram of the plasmonic system with single-cavity-coupled is illustrated in Fig 1(A). As shown in this two-dimensional mode sketch, a discrete MIM waveguide is side-coupled with a disk cavity with a gap of d. The width of the MIM waveguide is W, the gap distance is g and the radius of the disk is denoted by r. In our proposed structure, the nanodisk cavity and slit waveguide in white is assumed as air with refractive index 1, and the background in blue is silver.

The dispersion relation of the fundamental TM mode in an MIM waveguide is given by[20]

$$\varepsilon_{in}k_{z2}+\varepsilon_{m}k_{z1}\;\coth(ik_{z1}d/2)=0$$ (1)

with k_z1_ and k_z2_ defined by momentum conservations:

$$k_{z1}^{2}=\varepsilon_{in}k_{0}^{2}-\beta^{2},\;k_{z2}^{2}=\varepsilon_{m}k_{0}^{2}-\beta^{2}.$$ (2)

Where ε_in_ and ε_m_ are dielectric constants of the insulator and the metal respectively, *k*_0_ = 2*π*/*λ*_0_ is the free-space wave vector. The propagation constant β is represented as the effective index *n*_*eff*_ = *β*/*k*_0_ of the waveguide for SPPs.

The dielectric constant of the metal silver is characterized by the Lorentz–Drude model[21]

$$\varepsilon_{m}(\omega )=\varepsilon_{\infty }-\frac{\omega_{p}^{2}}{\omega^{2}+i\omega \gamma }$$ (3)

Here ε_∞_ = 3.7 is the relative permittivity in the infinity frequency, ω_p_ = 9.1eV, and γ = 18meV.

The exciting stable standing wave in the disk resonator forms the resonant condition, which can be given by[22]

$$k_{d}\frac{H_{n}^{(1)'}(k_{m}r)}{H_{n}^{\left(1\right)}(k_{m}r)}=k_{m}\frac{J_{n}^{'}(k_{d}r)}{J_{n}(k_{d}r)}$$ (4)

Here *k*_*d*,*m*_ = *k*(*ε*_*d*,*m*_)^1/2^ are the wave vectors in the metal and the dielectric nanodisk, respectively. ε_m_ stands for the relative dielectric constant of the metal, which is obtained from Lorentz-Drude model in Eq (3). ε_d_ is the effective permittivity of the dielectric. ${k=k}_{0}\sqrt{\frac{\varepsilon_{m}\varepsilon_{d}}{\varepsilon_{d}+\varepsilon_{m}}}$ stands for the wave number which includes a relatively small negative imaginary part standing for the loss. r represents the radius of the nanodisk cavity. $H_{n}^{\left(1\right)}$ and $H_{n}^{(1)'}$ are the Hankel function with the order n and its derivation, respectively. *J*_*n*_ and $J_{n}^{'}$ are the Bessel function with the order n and its derivation, respectively. The first and second order of Bessel and Hankel functions correspond to the first and second order modes that resonate inside. From Eq (4) one can find that the resonance wavelength λ is determined by effective refractive index n_eff_ and the radius r.

According to the Coupled mode theory[23,24], the transmission T of the system can be described as

$$T(\omega )=\frac{{\left(1/\tau_{\omega }\right)}^{2}}{{\left(\omega -\omega_{0}\right)}^{2}+{\left(1/\tau_{\omega }+1/\tau_{i}\right)}^{2}}$$ (5)

Where *ω* is the frequency of the incident light, 1/*τ*_*i*_ is the decay rates of internal loss in the resonators and 1/*τ*_*ω*_ is the decay rate of the energy escaping into the waveguides. We neglected the intrinsic loss (1/*τ*_*i*_ << 1/*τ*_*ω*_) and obtain *T*_max1_ = (1/*τ*_*ω*_)^2^/(1/*τ*_*ω*_ + 1/*τ*_*i*_)^2^ approximate to 1 when *ω* = *ω*_0_.

In the paper, the FDTD method with perfect matched layer and grid sizes 5 ⅹ 5 nm is adopted to investigate the characteristics of the plasmonic system. The simulated transmission spectra for the MIM waveguide with single cavity is shown in Fig 1(B). From it, we could see two resonant wavelengths with transmission 72.8% at 823nm and transmission 71% at 1320nm, respectively. The transmission peaks do not reach 1 for the waveguide loss and the internal loss in the disk cavity. This simulation result is consistent with the above-mentioned theory analysis.

## EIA-like response in dual-resonator-coupled system

**Fig 2:**
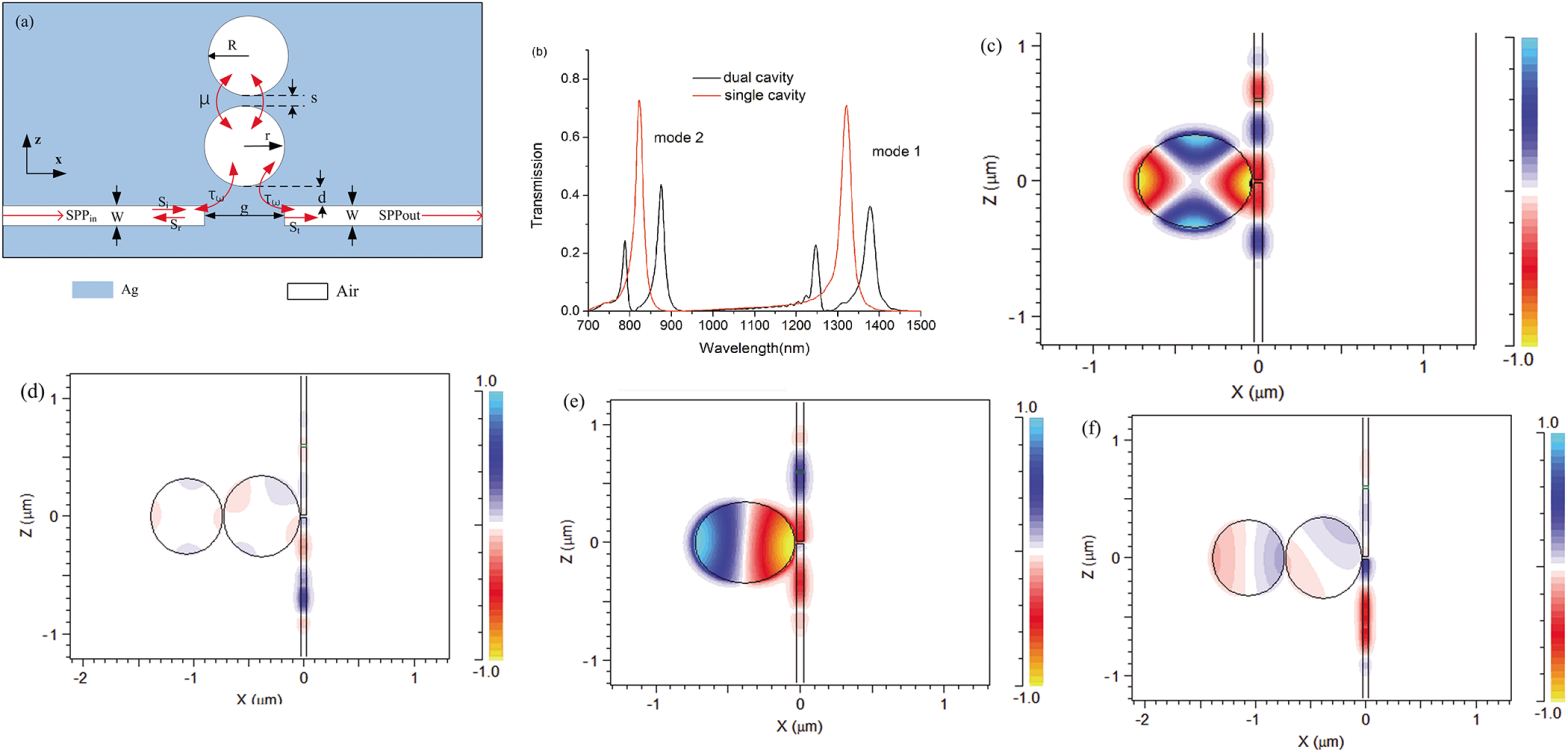
(a) Schematic of MIM waveguide side-coupled with dual disk cavities. (b) The transmission spectrum of the dual cavities structure with W = 50nm, d = 12nm, g = 30nm, s = 10nm, r = 345nm and R = 325nm. (c-f) Magnetic fields of the structure for monochromatic light at wavelength 823nm of single cavity system, 823nm of dual cavities system, 1320nm of single cavity system and 1320nm of dual cavities system, respectively.

Our proposed plasmonic system is composed of a discrete slit waveguide side-coupled with dual disk cavities, as shown in Fig 2(A). The transmission spectrum of the dual-cavities system is illustrated in Fig 2(B), with W = 50nm, d = 12nm, g = 30nm, s = 10nm, r = 345nm and R = 325nm. From Fig 2(B), we could see the transmission forms a dip around the transmission peak of the single cavity system in both mode 1 and mode 2. This phenomenon with pronounced absorption window is an EIA type spectral response, which is an opposite effect of EIT. In contrast with the single cavity system, the transmittance value decreases from 72.8% to 1.5% at the wavelength of 823nm and decreases from 71.0% to 2.9% at the wavelength of 1320nm, respectively. The absorption windows are due to the constructive interference between the two transmission paths, i.e. the excitation of resonant mode in the inside disk coupling from the incident wave in the MIM waveguide and the excitation coupling with the outside disk. The magnetic field distributions of resonant modes of the single cavity system and dual cavities system at 823nm and 1320nm are shown in Fig 2(C) to Fig 2(F), respectively. In Fig 2(C) and 2(E), one could see the *H*_*y*_ fields in the inside disk cavity is in-phase with the fields in the input MIM waveguides, thus the incident light and the light escaping into the bus waveguide from the inside disk has a coherence enhancement. On the contrary, in Fig 2(D) and 2(F), the *H*_*y*_ fields in the inside disk cavity is anti-phase with the fields in the input MIM waveguides, which leads to a resonance destructive and preventing the optical waves transmitting. Compared with the triple-cavity structure Meng et al.[25] proposed and the double-ring structure Wang et al.[19] prosed, the system we proposed is novel, simple and easy to be fabricated.

For characterizing the system by Coupled mode theory, as labeled in Fig 2(A), we define the following parameters: a and b is the cavity mode amplitude of two disk cavities, respectively; ω is the resonant frequency (wavelength); S_i_/S_r_/S_t_ are the incident/reflected /transmitted waveguide mode amplitudes, which are normalized and their squared values equal to incident/reflected /transmitted power; 1/τ_i_ and 1/τ_ω_ are decay rates due to intrinsic loss and waveguide coupling loss, respectively; μ is the coupling coefficient between the two disk cavities; $j=\sqrt{-1}$. The evolution of fields a and b can be described from the CMT

$$\frac{da}{dt}=(j\omega_{r}-\frac{1}{\tau_{i}}-\frac{2}{\tau_{\omega }})a+\sqrt{\frac{1}{\tau_{\omega }}}S_{i}+j\mu b$$ (6)

$$\frac{db}{dt}=(j\omega_{R}-\frac{1}{\tau_{i}})b+j\mu a$$ (7)

$$S_{t}=j\sqrt{\frac{1}{\tau_{\omega }}}a$$ (8)

According to Eq (6) to Eq (8), the transmission T of the output waveguide can be obtained as followed

$$\begin{array}{l} T={\left|\frac{S_{t}}{S_{i}}\right|}^{2}={\left|\frac{j}{j\tau_{\omega }(\omega -\omega_{r})+\frac{\tau_{\omega }}{\tau_{i}}+2+\frac{\beta^{2}\tau_{\omega }\tau_{i}}{j(\omega -\omega_{R})\tau_{i}+1}}\right|}^{2} \end{array}$$ (9)

When *ω* = *ω*_r_ = *ω*_*R*_, T can be simplified as $T={\left|\frac{j}{\frac{\tau_{\omega }}{\tau_{i}}+2+\beta^{2}\tau_{\omega }\tau_{i}}\right|}^{2}$, since *τ*_*ω*_ ≫ *τ*_*i*_, the value of T tends to zero.

**Fig 3:**
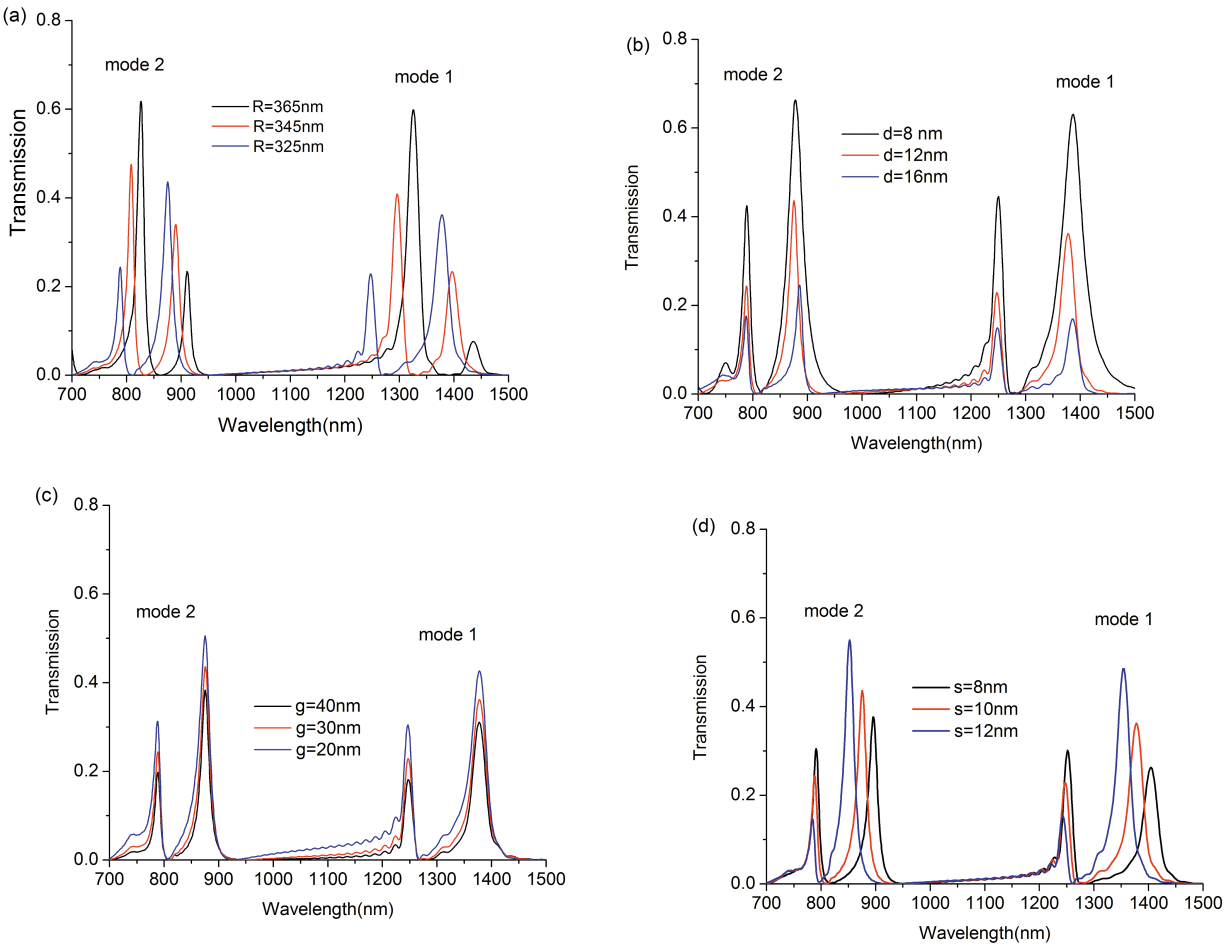
Transmission spectra for (a) different radii R of the outside cavity with W = 50nm, d = 12nm, g = 30nm, s = 10nm, r = 345nm. (b) different distance d between the inside disk cavity and MIM waveguide with W = 50nm, g = 30nm, s = 10nm, r = 345nm and R = 325nm. (c) different gap value g between the left and right part of the MIM waveguide with W = 50nm, d = 12nm, s = 10nm, r = 345nm and R = 325nm. (d) different distance s between the disk cavities with W = 50nm, d = 12nm, g = 30nm, r = 345nm and R = 325nm.

The transmission spectra of the proposed system with structure parameters changes are shown in Fig 3(A) to 3(D) respectively. From Fig 3(A), we could see the absorption windows have a red shift with the increasing of the radius of the outside disk cavity, which makes it possible to realize EIA-like response at selected wavelength by adjusting the radius of the disk. As shown in Fig 3(B), with d increasing from 8nm to 16nm in steps of 4nm, the transmission peak is reducing. This is due to the increase of coupling energy loss between the waveguide and the inside disk cavity. Fig 3(C) indicates the transmittance reduces a little with g increases from 20nm to 40nm. We can find the FWHM (the full width half maximum) changes with the variation of s in Fig 3(D).

## Conclusion

In summary, we have theoretically and numerically demonstrated an analog of double EIA based on a MIM waveguide side-coupled with dual disk cavities. The transmission properties of the proposed system were investigated by the FDTD methods. The FDTD simulation results agree well with the CMT. The absorption windows can be easily tuned by adjusting the geometrical parameters of the cavity. This work may be useful for EIA- like design and has potential applications in optical filtering, light switching, absorption, and sensing.

## Supporting information

S1 Data The detail simulation results data for Fig 1(B) in the manuscript. (OPJ)

S2 Data The detail simulation results data for Fig 2(B) in the manuscript. (OPJ)

S3 Data The detail simulation results data for Fig 3(A) in the manuscript. (OPJ)

S4 Data The detail simulation results data for Fig 3(B) in the manuscript. (OPJ)

S5 Data The detail simulation results data for Fig 3(C) in the manuscript. (OPJ)

S6 Data The detail simulation results data for Fig 3(D) in the manuscript. (OPJ)

S1 File The structure parameter setting source file of the dual-disk system proposed in the manuscript. (IND)
